# The impact of non-synonymous mutations on miRNA binding sites within the SARS-CoV-2 *NSP3* and *NSP4* genes

**DOI:** 10.1038/s41598-023-44219-y

**Published:** 2023-10-07

**Authors:** S. M. Ali Hosseini Rad, Dhammika Leshan Wannigama, Nattiya Hirankarn, Alexander D. McLellan

**Affiliations:** 1 Department of Microbiology and Immunology, University of Otago, Dunedin, New Zealand; 2 Center of Excellence in Immunology and Immune-Mediated Diseases, Chulalongkorn University, Bangkok, Thailand; 3 Department of Microbiology, Faculty of Medicine, Chulalongkorn University, King Chulalongkorn Memorial Hospital, Thai Red Cross Society, Bangkok, Thailand; 4 Department of Infectious Diseases and Infection Control, Yamagata Prefectural Central Hospital, Yamagata, Japan; 5 Center of Excellence in Antimicrobial Resistance and Stewardship Research, Faculty of Medicine, Chulalongkorn University, Bangkok, Thailand; 6 School of Medicine, Faculty of Health and Medical Sciences, The University of Western Australia, Nedlands, WA Australia; 7 Biofilms and Antimicrobial Resistance Consortium of ODA Receiving Countries, The University of Sheffield, Sheffield, UK; 8 Pathogen Hunter’s Research Team, Department of Infectious Diseases and Infection Control, Yamagata Prefectural Central Hospital, Yamagata, Japan; 9 Yamagata Prefectural University of Health Sciences, Kamiyanagi, Yamagata, 990-2212 Japan

**Keywords:** Biochemistry, Biotechnology, Genetics, Microbiology, Molecular biology, Diseases, Medical research

## Abstract

Non-synonymous mutations in the SARS-CoV-2 spike region affect cell entry, tropism, and immune evasion, while frequent synonymous mutations may modify viral fitness. Host microRNAs, a type of non-coding RNA, play a crucial role in the viral life cycle, influencing viral replication and the host immune response directly or indirectly. Recently, we identified ten miRNAs with a high complementary capacity to target various regions of the SARS-CoV-2 genome. We filtered our candidate miRNAs to those only expressed with documented expression in SARS-CoV-2 target cells, with an additional focus on miRNAs that have been reported in other viral infections. We determined if mutations in the first SARS-CoV-2 variants of concern affected these miRNA binding sites. Out of ten miRNA binding sites, five were negatively impacted by mutations, with three recurrent synonymous mutations present in multiple SARS-CoV-2 lineages with high-frequency *NSP3*: C3037U and *NSP4*: G9802U/C9803U. These mutations were predicted to negatively affect the binding ability of miR-197-5p and miR-18b-5p, respectively. In these preliminary findings, using a dual-reporter assay system, we confirmed the ability of these miRNAs in binding to the predicted *NSP3* and *NSP4* regions and the loss/reduced miRNA bindings due to the recurrent mutations.

## Introduction

microRNAs (miRNAs) are 18–24 bp non-coding RNAs that play a central role in the post-transcriptional regulation of gene expression. A single miRNA can target multiple genes simultaneously and alter cell growth, signalling or metabolic pathways. As such, dysregulation of miRNA expression have been linked to human diseases, including the severity of viral infections^1^. DNA and RNA viruses, including SARS-CoV-2, may produce miRNAs to manipulate the expression of host or viral genes to create an environment that favors viral replication^2, 3^.

Several studies indicate the interaction of the host miRNA system with viral genes. Such interactions may have a positive impact on viral replication by increasing translation caused by changes in RNA stability or RNA secondary structures^4, 5^. However, most miRNAs that interact with viral genes act as a defense mechanism to restrict viral replication. Such miRNAs must express in a physiologically relevant context. This means the miRNAs must be among cell/tissue-specific miRNAs. They also have to express a sufficient copy number with a strong complementary sequence beyond the seed sequence^2, 6^. These criteria are more important in the case of acute viral infections than chronic infections, where most viruses' life cycle is less than 12 h^6^. However, it should be noted that some miRNAs that are upregulated upon inflammatory responses, e.g., interferon stimulation, can impact viral replication in the adjacent cells that might lack the same miRNA expression pattern. Therefore, miRNAs are components of innate immunity targeting sequences in the viral genome to restrain viral replication in specific cell types. For instance, miR-142-3p binding sites within the eastern equine encephalitis virus (EEEV) result in the inability of EEEV to infect myeloid-lineage cells^7^. Interestingly, in a mouse model where the miRNAs binding sites (MBS) were mutated, EEV could replicate in macrophages^7^.

Several studies have attempted to predict host miRNA interaction with the SARS-CoV-2 genome. However, most of these studies rely on the sequence complementary matches and therefore identify mutliple putative binding miRNAs. In May 2020, using several independent programs, we identified ten miRNAs with a high complementary capacity to target various regions of the SARS-CoV-2 genome^8^. We filtered our candidate miRNAs to those only expressed with documented expression in SARS-CoV-2 target cells, with an additional focus on miRNAs that have been reported in other viral infections as components of the miRNA-mediated defense system. Next, we determined if these MBS were affected by mutations in the first SARS-CoV-2 variants of concern. Out of ten miRNA binding sites, five were negatively impacted by mutations, with three synonymous recurrent mutations present in multiple SARS-CoV-2 lineages with high-frequency *NSP3*: C3037U and *NSP4*: G9802U/C9803U. These mutations were predicted to negatively affect the binding ability of miR-197-5p and miR-18b-5p, respectively. Using a dual-reporter assay system, we confirmed the loss of miR-197-5p and miR-18b-5p binding to the mutated sequences within the *NSP3* and *NSP4* coding regions (CDS).

## Results

**Figure 1:**
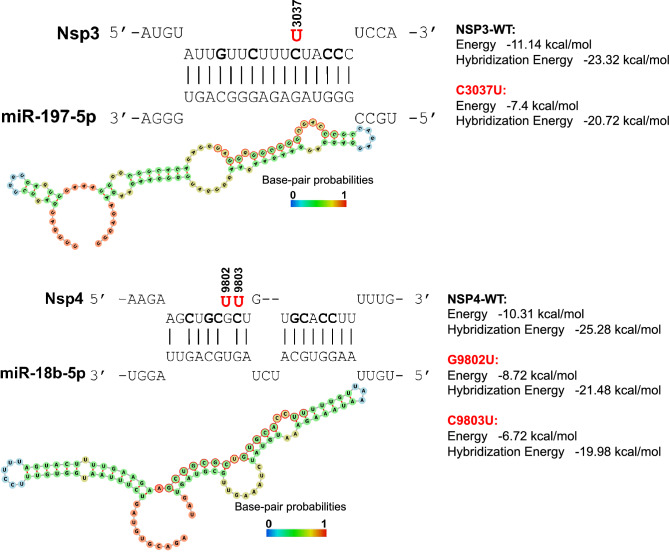
Prediction of miR-197-5p and miR-18b-5p binding sites within SARS-CoV-2 genome. The mutations that occur in miRNA binding sites (MBS) are indicated, and the designations of the mutations are shown in red font. The nucleotide substitutions that significantly affect MBS are shown in bold. The interaction figure and energy calculation were produced using the IntaRNA tool. Secondary RNA structures (based on minimum free energy (MFE) structure) of 100 bp surrounding the mutation sites (50 bp upstream and 50 bp downstream) with miRNA binding sites (red nucleotides) were created using the RNAfold program.

Several independent programs predicted the ability of miR-197-5p in binding to *NSP3* (nt: 3027–3042), and miR-18b-5p to the *NSP4* (nt: 9796–9813) (Fig. 1). These miRNAs are among the miRNAs expressed in the SARS-CoV-2 target cells and modulate the expression of dozen genes in target tissues (Supporting information S1, also see Ref.^8^). Structurally, these miRNAs bind to the accessible regions in the RNA secondary structure. Mapping nucleotides sensitive to substitutions revealed that C3037 and C9803 are among the nucleotides, that their substitution has a major negative impact on miRNA binding. In addition, such mutations resulted in the loss of miRNA binding prediction by software^8^. All three mutations cause decreased interaction energy between miRNAs and the binding sites (Fig. 1).

**Figure 2:**
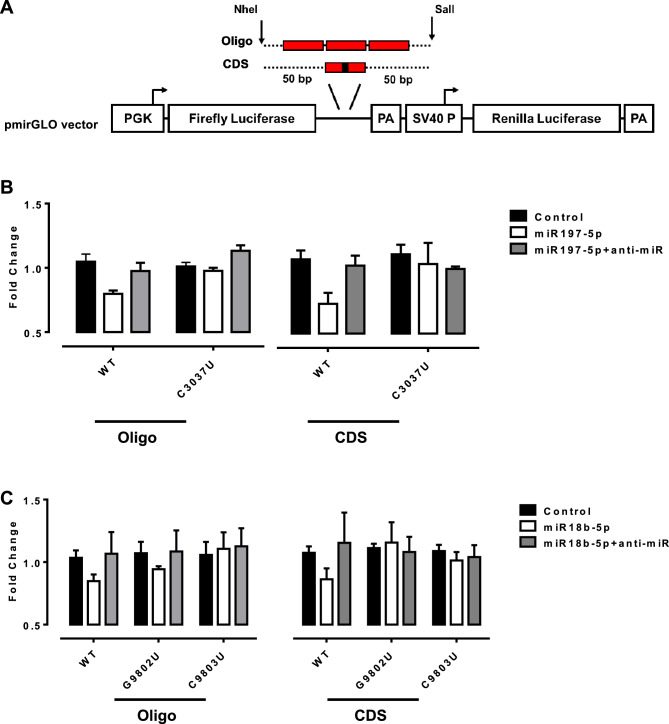
(**A**) Schematic representation of constructs used for reporter assay with wildtype (WT) or mutated sequence. MBS are shown in red boxes. (**B**,**C**) HEK293 cells were used for reporter assay experiments, and reporter assay was carried out 48 h post-transfection with the reporter vector alone (Control) or co-transfected with miRNA mimics and miRNA inhibitor. Luciferase assay was carried out by the Dual-Luciferase® Reporter assay system (Promega) and Varioskan LUX Multimode Microplate Reader read plates. RLU (relative light unit) from luc2 were divided to hRluc. For treat group, fold change was calculated by dividing the numbers from treat/control and for control group we divided the numbers from control to empty vector (with no Oligo/CDS insertion). Bar graph values represent the mean values ± SD from three independent repeats.

In order to further evaluate the ability of predicted miRNA in binding to their target regions, we used pmirGLO Dual-Luciferase vector (Promega), designed to quantitatively evaluate miRNA binding sites where miRNA binding sites (MBS) are downstream of Firefly luciferase gene (luc2) while the Renilla luciferase gene (hRluc) is expressed constitutively as an internal control (Fig. 2A). Generally, three MBS (Oligo) are inserted downstream of the Firefly gene to confirm an MBS. However, since our MBS are within the coding sequence and predicted MBS might not be accessible for RNA interactions, we also inserted 100 bp surrounding mutation site (CDS), which is a reasonable span length to accurately predict RNA secondary structure and accessibility^9^.

HEK293 cells were transfected with empty plasmids without MBS, plasmids carrying wildtype (WT) or mutated MBS (control group), MBS plasmids co-transfected with miRNAs. miRNA inhibitors (anti-miR) were also used to monitor the specificity of miRNAs by reversing the inhibitory effects. As Fig. 2B,C show, both miRNAs were able to bind to the predicted regions. Interestingly, one repeat of MBS within CDS had a similar inhibitory effect compared to three only MBS repeats in Oligo constructs. These data suggest the accessibility of predicted regions with strong binding capability for both miRNAs. In line with bioinformatic analysis, all three mutations were able to rescue the negative inhibitory effect of miRNAs (Fig. 2B,C). These data advocate that *NSP3*: C3037U and *NSP4*: G9802U/C9803U significantly reduce or abolish the binding ability of miR-197-5p and miR-18b-5p, respectively. All three mutations are synonymous and do not alter the secondary structure of RNA^8^. C3037U mutation was first identified in January 2020, while both G9802U/C9803U mutations were recognized in March 2020.

## Discussion

miR197-5p and miR-18b-5p are upregulated in patients with cardiovascular disease, a group of patients overrepresented in symptomatic COVID-19 cohorts with a higher mortality rate and have been reported to play a role in several viral infections^9–14^. miR-197-5p was reported to act as a defense mechanism against HBV, HCV, HAV, EBV and Enterovirus 71^15–19^ and its expression upregulated in serum during H7N9 influenza virus^20^. Similarly, altered expression of miR-18b-5p has been reported during several viral infections such as EBV, HBV, HCV, and Ebola^21–24^.

The exact mechanism of appearance of these synonymous recurrence mutations is unknown. We and others have noticed that most SARS-CoV-2 mutations are C/G → U substitution^8, 25^ and doubtful results of a replication-dependent process^26^. The majority of C → U mutations are likely the result of APOBEC RNA editing machinery, whereas G → U mutations are probably caused by reactive oxygen species (ROS) activity^25, 26^. The association of RNA editing machinery and miRNA RISC complex assembly is well established^27^. Therefore, it is possible that the interaction of miRNA to a viral gene facilitates the recognition and mutating of these regions by RNA editing machinery. This might also explain the high frequency and recurrence of these mutations.

Numerous studies have shown the interaction between viruses and host miRNA machinery. While a few viruses take advantage of the miRNAs to promote or regulate their replications, most of these interactions lead to the inability of a virus to infect certain cell types (e.g., miR-142-3p and EEV) or decrease viral replication. Coronaviruses have the largest genomes known (~ 30 kb) among RNA viruses. Therefore, it is not completely impossible that after switching to a new host, a few host miRNAs with 6–8 bp complementary regions may recognize the SARS-CoV-2 genome. In this preliminary finding, using strong bioinformatic evidence backed by reporter assays, we provided the first line of evidence showing the possibility of miR-197-5p and miR-18b-5p binding to the SARS-CoV-2 genome and the synonymous recurrence mutations that may have given the virus the advantage of escaping from the miRNA recognition. However, our study is limited due to not using a live virus. We do not have access and approval to the facilities to culture live virus nor early isolates of virus. Also, at the time of the experiments, we were unable to source commercial antibodies against nsp3 and nsp4 proteins for western blot or flow cytometry. Therefore, further studies must confirm our results using appropriate in vitro assays with live viruses.

## Methods

### Bioinformatic analysis

Computational analysis were performed as previously described in detail^8^. IntaRNA (one interaction per RNA pair, minimum 7 base pairs in seed, no seed with GU end, no lonely base pairs) was used to draw the interaction figure and energy calculation. For predicting crucial nucleotides within miRNA and target binding we used CopomuS (no A:U, G:U base pairs, no lonely base pairs, no helix ends, IntaRNA parameters: no GU at helix ends, min. 7 base pairs in seed). RNAfold program to predicted RNA secondary structures and base-pair probabilities based on minimum free energy (MFE) structure.

### Cloning

Three repeats of miRNA binding site (Oligo) 50 bp upstream + 50 downstream of the mutation sites (CDS) were cloned downstream of Firefly luciferase gene using NheI and SalI restricton enzymes in pmirGLO Dual-Luciferase vector (Promega).All sequences were synthesized by IDT and cloning was verified by sequencing. NSP3 and NSP4 CDS sequences with MBS highlighted in bold and mutated nucleotides underlined are listed below:

**NSP3 CDS:** TTT GAT GAG TCT GGT GAG TTT AAA TTG GCT TCA CAT ATG **TAT TGT TCT TTC TAC CCT** CCA GAT GAG GAT GAA GAA GAA GGT GAT TGT GAA GAA GAA GAG TT.

**NSP4 CDS:** AGA CGT GTA GTC TTT AAT GGT GTT TCC TTT AGT ACT TTT GAA GA**A GCT GCG CTG TGC ACC TT**T TTG TTA AAT AAA GAA ATG TAT CTA AAG TTG CGT AGT GAT.

### Cell culture, transfection, and luciferase assay

Human embryonic kidney (HEK) 293 T cells (Commercial available (HEK) 293 T cells were obtain from American Type Culture Collection-ATCC CRL-1573) were cultured in high glucose Dulbecco's Modified Essential Medium (DMEM) supplemented with 10% fetal bovine serum (FBS; Pan-Biotech GmbH), penicillin (100 U/mL), and streptomycin (100 μg/mL) (Gibco) at 37 °C, 5% CO_2_. For transfection, 3 × 104 HEK293T cells were seeded in 96-well plates without adding antibiotics. The next day, cells were transfected using lipofectamine 3000 (ThermoFisher) according to the manufacturer's protocol. Cells were either transfected with a 0.2 μg of reporter plasmid (Control group) or co-transfected with 5 nM of mirVana miRNA mimics and 50 nM of miRNA inhibitors (ThermoFisher). After 48 h post-transfection, luciferase activity was measured by adding reagents of the Dual-Luciferase Reporter assay system (Promega) and Varioskan LUX Multimode Microplate Reader read plates. RLU (Relative Light Unit) from luc2 were divided to hRluc. For treat group, fold change was calculated by dividing the numbers from control/treat/control and for control group we divided the numbers from control to empty vector (with no Oligo/CDS insertion).

### Ethical approval

The study did not involve human participants and was fully based on cell lines. Ethics approval was not required according to advice received from the Institutional Review Board (IRB) of the Department of Microbiology and Immunology, University of Otago, Dunedin, New Zealand.

### Supplementary Information

Supplementary Figures.

## Data Availability

All data underlying the results are available as part of the article and no additional source data are required.
