# Supplementary material for: The impact of non-synonymous mutations on miRNA binding sites within the SARS-CoV-2 NSP3 and NSP4 genes
Source: Sci Rep. 2023 Oct 7;13:16945. doi: 10.1038/s41598-023-44219-y (PMC10560223; doi:10.1038/s41598-023-44219-y)

Supplementary Figure 1

The expression level of miR-197-5p in normal human tissues based on **(A)** IMOTA database or **(B)** TISSUES. The numbers in the table represent the numbers of miR197-5-p targets in each tissues. The relative expression level of miR-197-5p is shown in blue or green color where the darker color shows higher expression level.

Image is created using IMOTA is an interactive multi-omics-tissue atlas and TISSUES with free to publish it under a CC BY open access license V. Palmieri, C. Backes, N. Ludwig, T. Fehlmann, F. Kern, E. Meese, A. Keller; IMOTA: an interactive multi-omics tissue atlas for the analysis of human miRNA–target interactions. Nucleic Acids Res 2018 gkx701, doi: 10.1093/nar/gkx701 and Developed by Alberto Santos, Oana Palasca, Christian Stolte, Kalliopi Tsafou, Sune Frankild, Janos Binder, Sean O'Donoghue, Jan Gorodkin, and Lars Juhl Jensen from the Novo Nordisk Foundation Center for Protein Research, Center for non-coding RNA in Technology and Health, and the Commonwealth Scientific and Industrial Research Organisation (CSIRO).

A

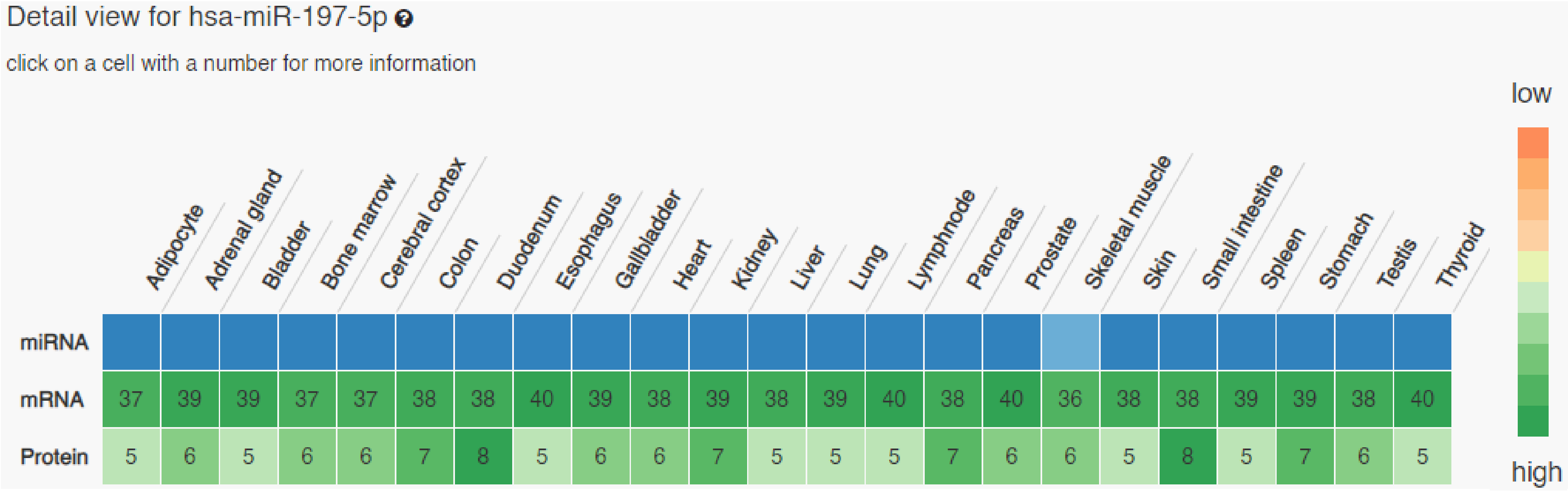

	Adipocyte	Adrenal gland	Bladder	Bone marrow	Cerebral cortex	Colon	Duodenum	Esophagus	Gallbladder	Heart	Kidney	Liver	Lung	Lymphnode	Pancreas	Prostate	Skeletal muscle	Skin	Small intestine	Spleen	Stomach	Testis	Thyroid
miRNA																							
mRNA	37	39	39	37	37	38	38	40	39	38	39	38	39	40	38	40	36	38	38	39	39	38	40
Protein	5	6	5	6	6	7	8	5	6	6	7	5	5	5	7	6	6	5	8	5	7	6	5

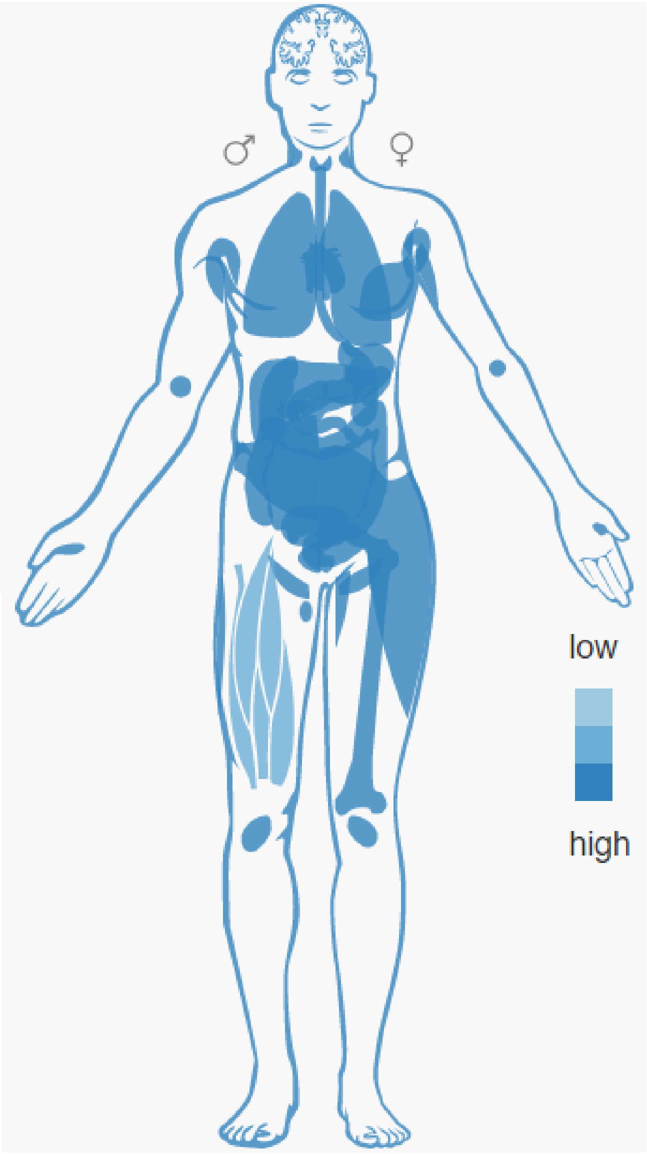

B

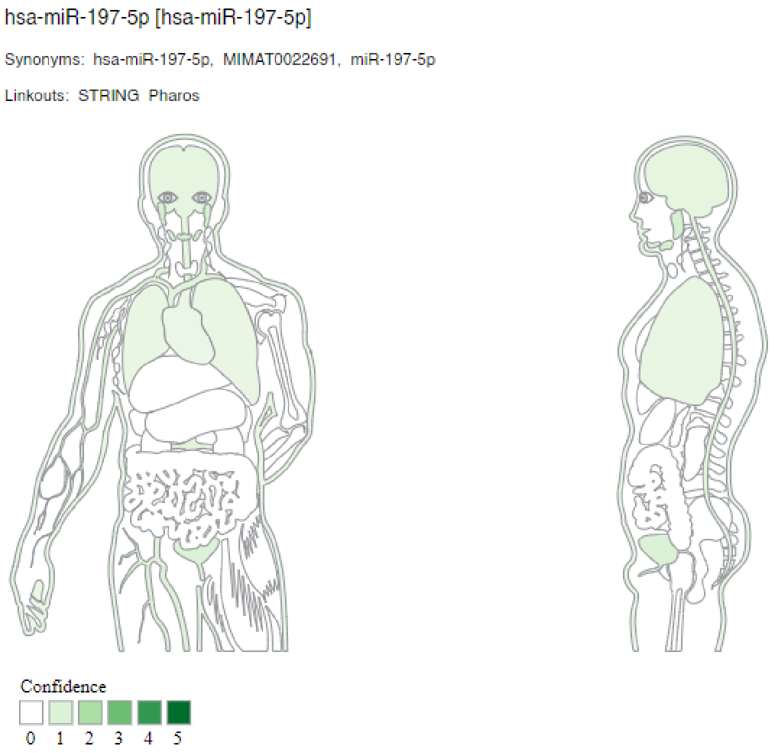

Supplementary Figure 2

The expression level of miR-18b-5p in normal human tissues based on **(A)** IMOTA database or **(B)** TISSUES. The numbers in the table represent the numbers of miR18b-5-p targets in each tissues. The relative expression level of miR-18b-5p is shown in blue or green color where the darker color shows higher expression level.

Image is created using IMOTA is an interactive multi-omics-tissue atlas and TISSUES with free to publish it under a CC BY open access license V. Palmieri, C. Backes, N. Ludwig, T. Fehlmann, F. Kern, E. Meese, A. Keller; IMOTA: an interactive multi-omics tissue atlas for the analysis of human miRNA–target interactions. Nucleic Acids Res 2018 gkx701, doi: 10.1093/nar/gkx701 and Developed by Alberto Santos, Oana Palasca, Christian Stolte, Kalliopi Tsafou, Sune Frankild, Janos Binder, Sean O'Donoghue, Jan Gorodkin, and Lars Juhl Jensen from the Novo Nordisk Foundation Center for Protein Research, Center for non-coding RNA in Technology and Health, and the Commonwealth Scientific and Industrial Research Organisation (CSIRO).

A

Detail view for hsa-miR-18b-5p

click on a cell with a number for more information

	Adipocyte	Adrenal gland	Bladder	Bone marrow	Cerebral cortex	Colon	Duodenum	Esophagus	Gallbladder	Heart	Kidney	Liver	Lung	Lymphnode	Pancreas	Prostate	Skeletal muscle	Skin	Small intestine	Spleen	Stomach	Testis	Thyroid
miRNA																							
mRNA	102	101	103	93	103	102	99	103	102	98	102	99	103	101	93	104	87	101	100	103	102	103	103
Protein	17	24	23	22	23	23	22	23	22	22	23	21	21	19	24	21	19	23	22	20	24	23	22

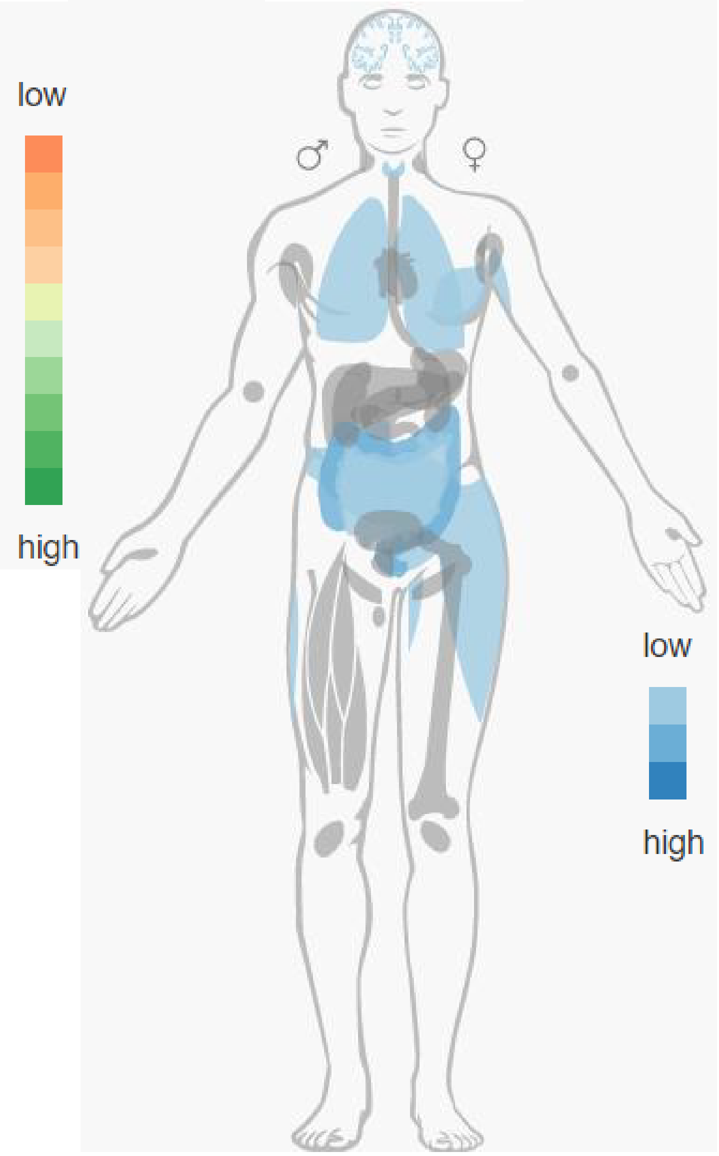

B

hsa-miR-18b-5p tissues

hsa-miR-18b-5p [hsa-miR-18b-5p]

Synonyms: hsa-miR-18b-5p, miR-18b-5p, hsa-miR-18b, MIMAT0001412, miR-18b

Linkouts: STRING Pharos

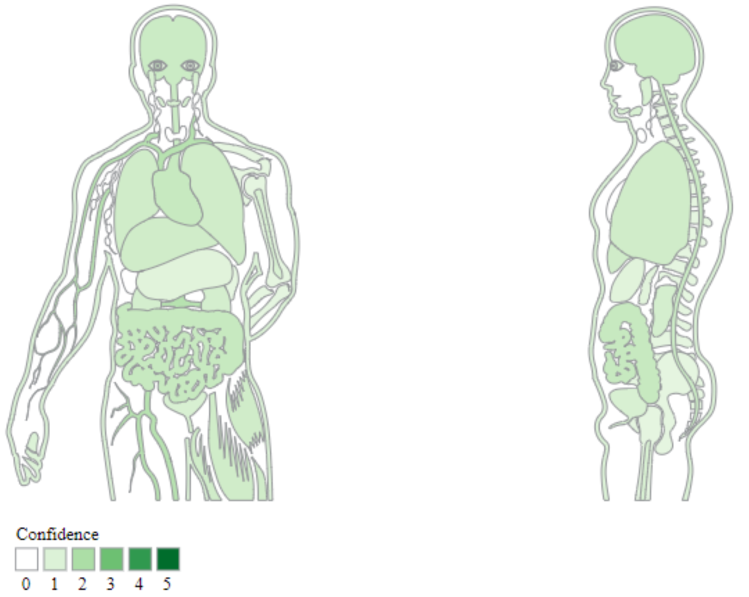

Supplement: Supplementary file 1 — Supplementary Figures. [file 41598_2023_44219_MOESM1_ESM.pdf]
